# The Efficacy of the Partial Glossectomy for Prevention of Airway Volume Reduction in Orthognathic Surgery of Class III Patients

**DOI:** 10.3390/life13020280

**Published:** 2023-01-19

**Authors:** Suyun Seon, Junho Jung, Baek-Soo Lee, Yong-Dae Kwon, Byung-Joon Choi, Joo-Young Ohe

**Affiliations:** Department of Oral and Maxillofacial Surgery, School of Dentistry, Kyung-Hee University, Seoul 02447, Republic of Korea

**Keywords:** pharyngeal airway, glossectomy, mandibular setback surgery

## Abstract

The aim of this study was to evaluate the effects of a partial glossectomy on volumetric changes of pharyngeal airway space (PAS) in patients with mandibular setback surgery. Overall, 25 patients showing clinical features related to macroglossia treated with mandibular setback surgery were included in this retrospective study. Subjects were divided into two groups: the control group (G1, *n* = 13, with BSSRO) and the study group (G2, *n* = 12, with both BSSRO and partial glossectomy). The PAS volume of both groups was measured by the OnDemand 3D program on CBCT taken shortly before operation (T0), 3 months post-operative (T1), and 6 months post-operative (T2). A paired t-test and repeated analysis of variance (ANOVA) were used for statistical correlation. Total PAS and hypopharyngeal airway space were increased after operation in Group 2 compared to Group 1 (*p* < 0.05), while oropharyngeal airway space showed no significant statistical difference with the tendency of increasing. The combination of partial glossectomy and BSSRO surgical techniques had a significant effect on increasing the hypopharyngeal and total airway space in class III malocclusion patients (*p* < 0.05).

## 1. Introduction

It is well known that orthognathic surgery on patients with skeletal malocclusion changes orofacial skeletal and soft tissues. Consideration of not only skeletal changes but also consideration of soft tissue changes are also emphasized after surgery because changes in location of skeletal and soft tissues rely on the mutual relationship between skeletal and neuromuscular components [1,2]. Alternation of soft tissues changes how they look but also changes the location of the tongue, location of the hyoid bone, and the pharyngeal airway space [3]. Among soft tissue components, it is well known that the size and location of the tongue affects the skeletal shape and teeth alignment, especially in those that have the skeletal class Ⅲ malocclusion. Macroglossia is not only a common cause of open bite and mandibular prognathism but also a cause of increasing risk of post-operative relapse and decreasing skeletal stability after orthognathic surgery [4,5]. Reducing the tongue volume by partial glossectomy is recommended as a treatment for patients with open bite and macroglossia. 

The importance of the size and location of the tongue becomes more apparent when mandibular setback is planned because the reduced volume of the oral cavity is occupied more by the tongue even though it is normal size [6]. According to many studies, the partial glossectomy is not a necessary procedure because the hyoid bone and the base of the tongue move downward as a compensation mechanism after orthognathic surgery so that the upper respiratory airway is kept open and the oral cavity is less occupied by the volume of the tongue [7,8]. However, if the hyoid bone and the base of tongue fail to move downward, therefore reducing the patient’s oral cavity, the patient is likely to have discomfort. Because the relative increase in size of the tongue causes a higher chance of post-operative relapse, and a lesser amount of overjet and overbite, both the skeletal and soft tissue components must be considered together. This is because the volume of airway space that is determined by the location of the hyoid bone and tongue affects post-operative stability and relapse; therefore, those considerations will improve the stability and prevent the relapse.

Partial glossectomy is known not to cause movement and speech problems of the tongue [9,10]. However, there is no objective criteria for partial glossectomy following orthognathic surgery until recently. Partial glossectomy is used depending on the symptoms of patients and a subjective decision made by surgeons. Up to recently, two-stage surgery is performed when a partial glossectomy is required following mandibular setback surgery because of airway obstruction by the tongue edema and bleeding. However, as the orthognathic surgery technique and method of fixation developed, the problem of post-operative airway obstruction was resolved, and two-stage surgery could be conducted simultaneously. Thus, reducing the size of the tongue by partial glossectomy improves post-operative stability and induces adaptation of the tongue [11,12]. 

Generally, lateral cephalography is used to measure the volume of airway space, but it has some limitations, such as anatomical structural overlapping and two-dimensional analysis. In contrast, digitalized computed tomography (CT), using a three-dimensional analyzing program, provides more detailed analysis by detecting volumetric changes and avoiding the overlapping problem.

Conventional orthognathic surgery alone can become challenging when the aim is preserving airway space. Thus, additional procedures such as tongue reduction due to macroglossia may be alternatives to a more functional outcome. The purpose of this study was to observe three-dimensional volumetric changes of pharyngeal airway space using Cone-Beam CT (CBCT) and find out the necessity of a partial glossectomy by comparison between the patients who underwent simultaneous mandibular setback surgery and partial glossectomy, and the patients who only underwent mandibular setback surgery.

## 2. Materials and Methods

### 2.1. Patients

Twenty-five patients who were diagnosed with skeletal class III malocclusion and treated with maxillary Le Fort I osteotomy and mandibular bilateral sagittal split ramus osteotomy (BSSRO) at the Department of Oral and Maxillofacial surgery, Kyung-Hee University School of Dentistry from 2010 to 2014 were identified. The inclusion criteria were as follows: (1) maxillary advancement surgery by 0–5 mm and mandibular backward movement by 2–15 mm; (2) macroglossia-related clinical features described by Wolford and Cottrell in their study; (3) 6 months follow-up with post-operative CBCT; (4) no obstructive sleep apnea (OSA)-related symptoms. The patients were categorized into 2 groups. Group 1 consisted of 13 patients (mean age of 24 ± 3.19 years) who underwent Le Fort I osteotomy and mandibular BSSRO. Group 2 included 12 patients (mean age of 25 ± 6.89 years) who underwent the same surgery as Group 1 but also underwent partial glossectomy simultaneously. Post-operatively, occlusion of those 2 groups was stabilized by splint and they underwent exactly the same post-operative treatment.

### 2.2. Surgical Procedure

For mandibular surgery, semi-rigid fixation was conducted with a monocortical plate and three miniscrews per side. Following mandibular movement, the miniplate was fixed to maintain the internal gap between bones in order to prevent the displacement of the proximal segment. After making a reference point on the ascending ramus of the mandible, the distance was measured from the reference point to more than 3 points on brackets on the maxillary teeth before surgery to maintain the original position of the proximal segment. Intermaxillary fixation was placed for 2 weeks post-operatively. An opening exercise of the mouth was performed for 4 weeks after the fixation period. In Group 1, maxillary advancement was 0–5 mm (mean 1.9 mm) and mandibular setback was 6–15 mm (mean 8.4 mm), whereas maxillary advancement was 0–3 mm (mean 1.19 mm) and mandibular setback was 2–13 mm (mean 8.5 mm) in Group 2. There were no significant differences in the movement of maxilla and mandible in Group 1 and Group 2. 

**Figure 1:**
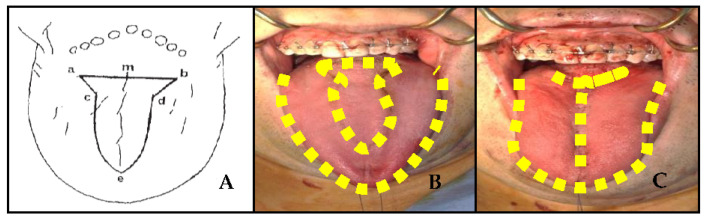
(**A**) Incision used for tongue reduction; (**B**) pre-operative; (**C**) post-operative.

We performed a T-shaped partial glossectomy that was recommended by Ueyama (9). The horizontal line (Figure 1A—a,b) at the dorsal surface of the tongue begins in front of the vallate papilla, and the tip portion (Figure 1A—e) begins at the dorsal surface of the tongue at least 1 cm from the apex of tongue. The incision is made in a horizontal line down to the upper part of the transverse lingual muscle, and then another incision is made in a V shape to eliminate any in the transverse lingual muscle. In order to eliminate the dead space and gather all parts of tongue in the center, inner muscles and surface tissues of the tongue were sutured. As a result, the length and width of the tongue were reduced. The reduced volume of the tongue was 7–22 cc (mean 9.91 cc). There were no patients complaining about movement, taste, or speech problems. 

The panoramic mode of CBCT was used to analyze the pre-operative (T0), 3 months post-operative (T1), and 6 months post-operative state (T2). The volume of the airway right after surgery was not measured because the nasopharyngeal airway was used for 3 days to keep the airway open, and it was significantly reduced due to edema of the soft palate, and edema coming from anesthesia and the surgical procedure on the wall of the pharyngeal airway. The CBCT that was used for measurement was the Vega 3030 Dental CT system (Asahi Roentgen Ind. Co., Ltd., Kyoto, Japan). The patient’s head was fixed to make the FH plane parallel to the floor and the shot was made in panoramic mode. All pictures were taken at 80 kVp and 5–10 mA with a duration of 17 sec. This study was approved by the Institutional Review Board (IRB) at Kyung-Hee University, School of Dentistry (KHD IRB 1510-2).

### 2.3. Volumetric Analysis

Analyzation was conducted with a 0.3 mm thickness of Raw-Dicom file, and OnDemand 3D (Cyber Med., Seoul, Republic of Korea) was used to analyze three-dimensionally. The analyzation was conducted by only one person to eliminate measurement errors and collected information was analyzed by OnDemand 3D that was used to measure the volume of airway at T0, T1, and T2. 

To measure the volume of the airway space, it was measured in 3 different ways as follows:

(1) Oropharyngeal airway space: the space was defined from the line which was passing through the posterior nasal spine and parallel to the FH plane to the end of the epiglottis, generally the most inferior portion of the soft palate.
(2) Hypopharyngeal airway space: the space was defined below the oropharyngeal airway space and up to the line which was passing through the epiglottis and parallel to the FH plane, generally at the level of the end of the 3rd cervical vertebra.
(3) Total airway was the sum of oropharyngeal airway space and hypopharyngeal airway space.

**Figure 2:**
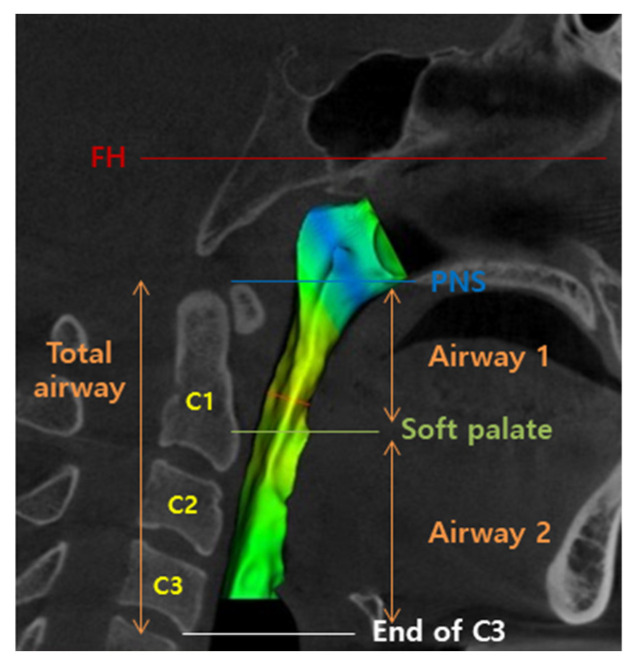
Demonstration of pharyngeal airway section with the volumetric analysis on the CT.

**Figure 3:**
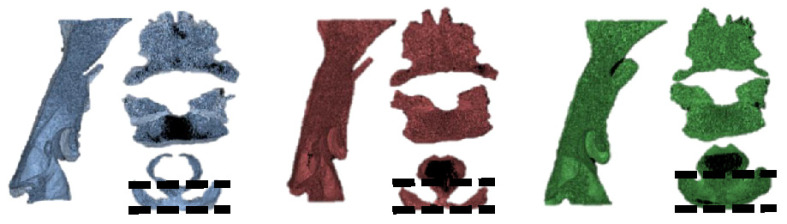
Axial view of airway space shows the increase of airway volume is related with the anterior-posterior width.

For easier understanding in this study, oropharyngeal airway space was renamed as “Airway 1” and hypopharyngeal airway space was renamed as “Airway 2” (Figure 2 and Figure 3).

### 2.4. Statistical Analysis

Mean and standard deviation were obtained for each volumetric change in airway at T0, T1, and T2 in both Group 1 and 2. To find out statistical correlation, SPSS v21.0 (IBM Co., Armonk, NY, USA) was used to perform a matched paired t-test. The statistical correlation with and without partial glossectomy was determined using repeated measure analysis of variance (ANOVA). To test their significance, each test was conducted at a significance level of 95%. 

## 3. Results

### 3.1. Volumetric Changes in Airway 1

**Figure 4:**
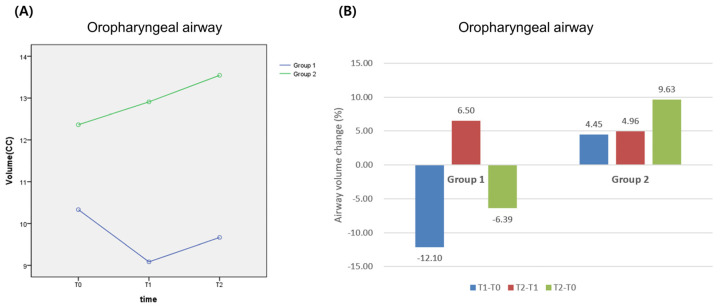
Change of oropharyngeal airway before and after surgery: (**A**) volumetric change; (**B**) percentage change. Group 1: Le Fort I + B-SSRO; Group 2: Le Fort I + B-SSRO + partial glossectomy.

Volume of Airway 1 in Group 1 was recorded as 10.33 cc at T0, 9.08 cc at T1, which was slightly reduced from T0 (*p* > 0.05), and 9.67 cc at T2, which showed a tendency of recovering but was still less than volume at T0 (*p* > 0.05). Volume of Airway 1 in Group 2 was recorded as 12.36 cc at T0, 12.91 cc at T1, which showed a tendency of recovering (*p* > 0.05), and 13.55 cc at T2 (*p* > 0.05), which was increased by 1.10 cc compared to volume at T0 (Table 1 and Table 2) (Figure 4). When a repeated measure ANOVA was used to find and analyze the volumetric changes in both Group 1 and 2, there was no statistically significant difference depending on time (*p* > 0.05). When the effect of both time and partial glossectomy was analyzed, there was no statistically significant difference (*p* > 0.05). 

### 3.2. Volumetric Changes in Airway 2

**Figure 5:**
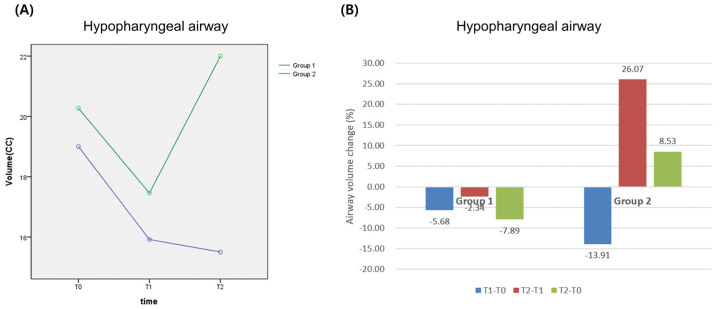
Change of hypopharyngeal airway before and after surgery: (**A**) volumetric change; (**B**) percentage change. Group 1: Le Fort I + B-SSRO; Group 2: Le Fort I + B-SSRO + partial glossectomy.

The volume of Airway 2 in Group 1 was recorded as 19.00 cc at T0, 17.92 cc at T1, which was slightly reduced (*p* > 0.05), and 17.50 cc at T2 (*p* > 0.05), which was reduced by 1.19 cc compared to volume at T0 but not significantly different (*p* > 0.05). The volume of Airway 2 in Group 2 was measured as 20.27 cc at T0, 17.45 cc at T1, which was reduced (*p* > 0.05), and 22.00 cc at T2, which showed a tendency of recovering (*p* < 0.05) and was increased by 1.65 cc compared to volume at T0 (*p* > 0.05) (Table 3 and Table 4) (Figure 5). When the differences in both groups were analyzed using a repeated measure ANOVA, there were statistically significant differences depending on the amount of recovery time after surgery (*p* < 0.05); therefore, the effect of partial glossectomy was proved (*p* < 0.05) (Table 5).

### 3.3. Volumetric Changes in Total Airway

**Figure 6:**
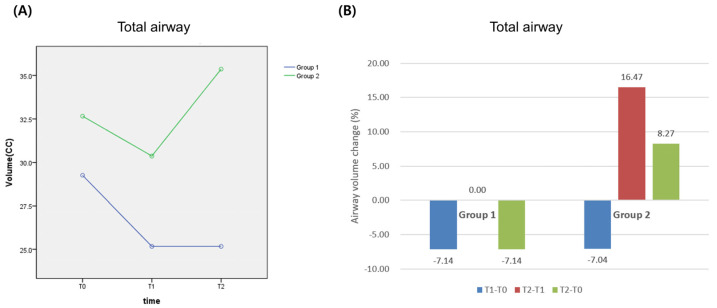
Change of total airway before and after surgery: (**A**) volumetric change; (**B**) percentage change. Group 1: Le Fort I + B-SSRO; Group 2: Le Fort I + B-SSRO + partial glossectomy.

The volume of the total airway in Group 1 was measured as 29.26 cc at T0, 27.17 cc at T1 (*p* > 0.05), which was reduced from T0 and 27.17 cc at T2, which was not changed from T1. It was reduced by 2.18 cc compared to the volume at T0, and there was no statistical difference (*p* > 0.05). The volume of total airway in Group 2 was recorded as 32.66 cc at T0, 30.66 cc at T1, which was reduced from T0, and 35.36 cc at T2, which showed a tendency of recovering by 2.75 cc. It showed a statistically significant difference compared from volume at T0 to volume at T2 (*p* < 0.05) (Table 6 and Table 7) (Figure 6). When the differences in both groups were analyzed by a repeated measure ANOVA, there was statistically significant difference in terms of time; the effect of partial glossectomy was proved (*p* < 0.05). This tendency of change was similar to the tendency of volumetric changes in the hypopharyngeal airway (Table 8).

## 4. Discussion

There have been many studies conducted about complications of orthognathic surgery on patients with skeletal class III malocclusion [13,14]. Furthermore, a large number of them are related to the study of changes in airway space after surgery, and it is an actively ongoing subject of research [15,16]. Gu et al. reported that there was a correlation in the location of the hyoid bone, volume of airway, and the position of the head after mandibular setback surgery, and compensation of the volumetric decrease in the airway caused a change in position of the hyoid bone and tongue [17]. Furthermore, Takaki et al. reported that tongue muscles moved downward due to the downward movement of the hyoid bone after mandibular setback surgery. This movement was a type of compensation for reduced airway space because of the downward movement of the tongue. As time passed, the hyoid bone had a tendency to go back to its original position. Moreover, the volume of airway space recovered to its original state [18]. However, these results are contradicted by some other studies [19,20]. These days, there are many studies about obstructive sleep apnea (OSA) after mandibular setback surgery [21,22]. Riley et al. reported that there was a higher risk of getting OSA after orthognathic surgery on patients with mandibular prognathism [23]. Hochban et al. also stated that OSA was induced if pharyngeal airway space after surgery was less than 10 mm. In this case, Hochban mentioned that orthognathic surgery, including maxillary advancement, had to be considered [24].

Especially, patients with mandibular prognathism and pseudomacroglossia have a higher risk of having a post-operative relapse, and this condition is one of factors that decrease skeletal stability [4,5]. Additionally, a decrease in airway space due to the reduced volume of oral cavity increases the chances of having patients feel discomfort after surgery. However, the effect of partial glossectomy on reducing discomfort is still controversial. Wickwire and Sinclare et al. mentioned that partial glossectomy was not required after surgery because pharyngeal airway space was maintained by downward movement of the tongue base and hyoid bone. Furthermore, the volume of the tongue was reduced in the recovering process [7,8]. In contrast, Ingervall et al. mentioned that as the mandible moved backward and the tongue also moved backward, total efficiency of breathing was decreased due to compression of the upper airway space. As the mandible moved downward to compensate for the increased intra-oral cavity pressure, post-operative stability was decreased, inducing anterior open bite [25]. Swanson and Petdachai et al. also insisted that as an enlarged tongue after orthognathic surgery pushed the mandible and anterior teeth of mandible, post-operative relapse could be caused [11,12]. Allison stated that partial glossectomy could help patients to reduce discomfort if the pre-mentioned compensation was not present and if there was an enlarged tongue right after surgery with no compensation yet [11].

Up to recently, there have been a lot of studies conducted about volumetric changes in airway space post-operatively using lateral cephalography and some reference points. However, the analysis was only able to be conducted two-dimensionally on pharyngeal airway space and the hyoid bone, such as the anterior-posterior relationship of airway space and the upward-downward movement of hyoid bone. Therefore, in this study, three-dimensional CBCT was used to analyze the effect of partial glossectomy on hypopharyngeal airway space changes of patients who underwent simultaneous partial glossectomy and mandibular setback surgery. In Group 1 without partial glossectomy, there was no statistically significant decrease in oropharyngeal and hypopharyngeal airway space even though there was a continuous decrease in airway space from T0 to T2. These results supported the study of Wickwire and Athanasious et al. They stated that as the mandible and its surrounding structures moved backward, the airway was expected to be compressed. However, functional and structural adaptation to compensate for airway compression made no significant change in volumetric airway space before and after surgery [7,26]. 

In Group 2 with partial glossectomy, there was no statistically significant changes in the volume of the oropharyngeal airway, but it showed slightly the tendency of increasing. In Group 1, the hypopharyngeal and total airway were reduced continuously from T0 to T2. In contrast, the hypopharyngeal and total airway were reduced from T0 to T1 but increased from T1 to T2 in Group 2. When the volumetric change of the pharyngeal airway was analyzed dependent upon post-operative time, there was a statistically significant difference in the hypopharyngeal and total airway, and it was thought to be the effect of a partial glossectomy. Since the volumetric change pattern in the hypopharyngeal was similar to the one in the total airway, it was thought to be that partial glossectomy increased hypopharyngeal airway space, and this change increased the total airway space. This result supports the studies of Kwakami that there were differences in the location of the base of the tongue between the patients who received and who did not receive a glossectomy [27]. It was not only the same as the result of Group 1, which was the result of recovering to its original volume of the airway, but also the effect of a partial glossectomy. In other words, decreasing the tongue size caused a decrease in clock-wise rotational movement of the mandible, and a decrease in the amount of invasion into the pharyngeal airway space by the tongue and its surrounding tissues. The reasons of volumetric changes in the hypopharyngeal and total airway of Group 2 from T0 to T2 are that the hyoid bone moves downward to compensate for the decrease in the volume of the oropharyngeal airway due to the backward movement of the mandible after surgery. Then, the movement of the hyoid bone causes a decrease in the volume of the hypopharyngeal airway. However, after 6 months since surgery, the hyoid bone moves back to its original position, and the tongue becomes stable in its size and location. Considering the results of this study, a partial glossectomy is considered to be effective for increasing the airway space with mandibular setback surgery.

Further studies are required on the effect of a partial glossectomy dependent on the shape of the tongue, the size of the tongue, the location of the tongue, the volume of airway space, and the amount of movement of the mandible. 

Swallowing and articulation disorders have been reported to be common complications of glossectomy [28]. The factors that influence their development are the extent and location of surgical resection and the flexibility of the residual tongue. Along with many other reports of partial glossectomy resulting in good post-operative tongue function, our post-operative follow-up showed that the function of swallowing and articulation over the long term is generally acceptable after a partial glossectomy.

While this study was focused on the efficacy of the partial glossectomy for prevention of airway reduction, there is currently insufficient direct data to draw any firm conclusions due to the small sample size and lack of post-operative assessment on long-term stability. Furthermore, some potential therapy using low-level lasers and Diode lasers for wound healing and pain relief have been reported [29,30]. Further studies to evaluate the treatment of lasers in prevention of complications of glossectomy in patients with orthognathic surgery are recommended.

## 5. Conclusions

According to the results of the study, it was concluded that:

1. There was decrease in the volume of airway space in the group without partial glossectomy, but the extent was not statistically significant.

2. In the group with partial glossectomy, their airway spaces were decreased from pre-operation to 3 months post-operative, but it was increased after 3 months. This was because the location of the hyoid bone and tongue muscle was recovered after 3 months post-operative, and partial glossectomy was considered as one of the causes that increased the volume of the oral cavity.

3. Based on the length of time following surgery, the tendency of volumetric changes in the oropharyngeal and total airway space of both groups revealed statistical differences. The reason for this change is that a partial glossectomy increased both hypopharyngeal and the total airway space. The essential clinical factors, such as post-operative stability, relapse prevention, and maintaining an open airway, were all improved by those changes [9,31].

In conclusion, according to the results regarding the tendency of volumetric changes in airway space of patients with skeletal class III malocclusion post-operatively, simultaneous mandibular setback surgery with partial glossectomy was determined to be helpful for patients who need backward movement of the mandible or who have high possibility of having respiratory obstructions such as snoring and obstructive apnea (OSA) after surgery.

## Figures and Tables

**Table 1:** Volume of oropharyngeal airway (CC).

	T0	T1	T2
	(Mean ± SD)	(Mean ± SD)	(Mean ± SD)
Group 1	10.33 ± 3.63	9.08 ± 2.68	9.67 ± 3.08
Group 2	12.36 ± 2.69	12.91 ± 2.88	13.55 ± 3.36

T0: pre-operation; T1: 3 months after operation; T2: 6 months after operation.

**Table 2:** Change of oropharyngeal airway before and after surgery (CC).

	T0–T1	T1–T2	T2–T0
	(Mean ± SD)	(Mean ± SD)	(Mean ± SD)
Group 1	1.48 ± 0.74	0.79 ± 1.28	−2.27 ± 1.57
Group 2	−0.54 ± 0.52	0.79 ± 1.28	1.10 ± 0.89

The results of matched paired t-test. T0: pre-operation; T1: 3 months after operation; T2: 6 months after operation.

**Table 3:** Volume of hypopharyngeal airway (CC).

	T0	T1	T2
	(Mean ± SD)	(Mean ± SD)	(Mean ± SD)
Group 1	19.00 ± 7.92	17.92 ± 5.63	17.50 ± 6.53
Group 2	20.27 ± 7.77	17.45 ± 7.69	22.00 ± 7.25

T0: pre-operation; T1: 3 months after operation; T2: 6 months after operation.

**Table 4:** Change of hypopharyngeal airway before and after surgery (CC).

	T0–T1	T1–T2	T2–T0
	(Mean ± SD)	(Mean ± SD)	(Mean ± SD)
Group 1	3.73 ± 1.35	1.79 ± 1.62	−1.19 ± 2.42
Group 2	2.82 ± 1.70	−4.48 ± 1.58 *	1.65 ± 1.69

* Indicates the significant variation of the matched paired t-test (*p* < 0.05). T0: pre-operation; T1: 3 months after operation; T2: 6 months after operation.

**Table 5:** Repeated measure analysis of variance between measurements of hypopharyngeal airway.

Time	Time × Glossectomy
0.012 *	0.013 *

* Indicates the significant variation (*p* < 0.05).

**Table 6:** Volume of total airway (CC).

	T0	T1	T2
	(Mean ± SD)	(Mean ± SD)	(Mean ± SD)
Group 1	29.26 ± 11.15	27.17 ± 7.98	27.17 ± 9.06
Group 2	32.66 ± 10.01	30.36 ± 9.86	35.36 ± 9.97

T0: pre-operation; T1: 3 months after operation; T2: 6 months after operation.

**Table 7:** Change of total airway before and after surgery (CC).

	T0–T1	T1–T2	T2–T0
	(Mean ± SD)	(Mean ± SD)	(Mean ± SD)
Group 1	1.08 ± 1.89	2.58 ± 2.84	−2.18 ± 3.92
Group 2	2.28 ± 2.01	−5.04 ± 2.49 *	2.75 ± 2.37 *

* Indicates the significant variation of the matched paired t-test (*p* < 0.05). T0: pre-operation; T1: 3 months after operation; T2: 6 months after operation.

**Table 8:** Repeated measure analysis of variance between measurements of Total airway.

Time	Time × Glossectomy
0.049 *	0.037 *

* Indicates the significant variation (*p* < 0.05).

## Data Availability

Data supporting reported results can be provided upon reasonable request.
